# Hospital Acquired Pneumonia Due to *Achromobacter* spp. in a Geriatric Ward in China: Clinical Characteristic, Genome Variability, Biofilm Production, Antibiotic Resistance and Integron in Isolated Strains

**DOI:** 10.3389/fmicb.2016.00621

**Published:** 2016-05-09

**Authors:** Chao Liu, Fei Pan, Jun Guo, Weifeng Yan, Yi Jin, Changting Liu, Long Qin, Xiangqun Fang

**Affiliations:** ^1^Intensive Care Unit, Beijing Haidian Hospital, Haidian Section of Peking University Third HospitalBeijing, China; ^2^Department of Gastroenterology and Hepatology, Chinese PLA General HospitalBeijing, China; ^3^Department of Respiratory Medicine, Beijing Tsinghua Changgung Hospital, Medical Center, Tsinghua UniversityBeijing, China; ^4^Nanlou Respiratory, Diseases Department, Chinese PLA General HospitalBeijing, China

**Keywords:** hospital-acquired pneumonia, *Achromobacter*, biofilm, elderly, integron

## Abstract

**Background:** Hospital-acquired pneumonia (HAP) due to *Achromobacter* has become a substantial concern in recent years. However, HAP due to *Achromobacter* in the elderly is rare.

**Methods:** A retrospective analysis was performed on 15 elderly patients with HAP due to *Achromobacter* spp., in which the sequence types (STs), integrons, biofilm production and antibiotic resistance of the *Achromobacter* spp. were examined.

**Results:** The mean age of the 15 elderly patients was 88.8 ± 5.4 years. All patients had at least three underlying diseases and catheters. Clinical outcomes improved in 10 of the 15 patients after antibiotic and/or mechanical ventilation treatment, but three patients had chronic infections lasting more than 1 year. The mortality rate was 33.3% (5/15). All strains were resistant to aminoglycosides, aztreonam, nitrofurantoin, and third- and fourth-generation cephalosporins (except ceftazidime and cefoperazone). Six new STs were detected. The most frequent ST was ST306. ST5 was identified in two separate buildings of the hospital. ST313 showed higher MIC in cephalosporins, quinolones and carbapenems, which should be more closely considered in clinical practice. All strains produced biofilm and had integron I and *bla_OXA-*114*-like_*. The main type was *bla_OXA-*114*q_*. The variable region of integron I was different among strains, and the resistance gene of the aminoglycosides was most commonly inserted in integron I. Additionally, *bla_PSE-*1*_* was first reported in this isolate.

**Conclusion:** *Achromobacter* spp. infection often occurs in severely ill elders with underlying diseases. The variable region of integrons differs, suggesting that *Achromobacter* spp. is a reservoir of various resistance genes.

## Introduction

*Achromobacter* is a ubiquitous, non-fermenting, gram-negative bacterium that lives in soil and aquatic environments. In recent years, many studies have shown that this opportunistic pathogen can lead to various infections, such as sepsis, bacteremia, urinary infection, endocarditis, and peritonitis, especially in immunocompromised individuals (Aisenberg et al., 2004). Moreover, this organism can colonize various medical devices, such as disinfectant-soaked unwoven cleaning cloth (Oie et al., 2012), intravascular pressure transducer (Gahrn-Hansen et al., 1988), and chlorhexidine (Vu-Thien et al., 1998).

To distinguish different *Achromobacte*r strains, various methods have been applied. Similar to OXA-50-like enzymes from *P. aeruginosa* or OXA-51/69 from *A. baumannii*, the *bla_OXA-*114*-like_* gene is a known characteristic of *Achromobacter* (Doi et al., 2008; Turton et al., 2011). Additionally, multilocus sequence typing (MLST) is one of the best methods for use in epidemiological studies in the world. There are more than 300 STs in the MLST database distributed across the world. However, due to the low incidence of *Achromobacter* infections, no clone complex has been reported.

Elderly patients often have underlying diseases and frequently suffer from chronic infections, which may result in continuous exposure to various types of antibiotics. This exposure may contribute to antibiotic resistance. Based on previous studies, integrons and the inborn RND-Type Multidrug Eﬄux Pump (Bador et al., 2011) are the main reasons for antibiotic resistance. Various integrons (intI/intII) have been detected (Traglia et al., 2012). Additionally, extended-Spectrum β-Lactamases (ESBL) and metallo-b-lactamase (MBL) production by *Achromobacter* spp. has been detected in the last decade. In Europe, *bla_V EB_* and *bla_TMB_* inserted in integrons were found in *Achromobacter* spp. (Neuwirth et al., 2006; El Salabi et al., 2012). Various subtypes of *bla_VIM_* have been detected in integron I in Asia and Europe (Riccio et al., 2001; Shin et al., 2005; Sofianou et al., 2005). Infections, pandemic or sporadic, are often related to the location of resistance genes (chromosome/plasmid). Thus, the insertion of a new *bla_IMP_*-type gene in plasmids in *A. xylosoxidans* has attracted wide attention because it may spread easily (Yamamoto et al., 2012). Moreover, the inherent *bla_OXA-*114*-like_* genes may result in a higher level of OXA-114-mediated-lactam resistance because of the insertion sequence elements (Doi et al., 2008). Therefore, *Achromobacter* spp. is an emerging pathogen and is becoming a reservoir for horizontal genetic transfer elements involved in spreading antibiotic resistance (Traglia et al., 2012).

Hospital-acquired pneumonia (HAP) due to *Achromobacter* spp. has emerged as a substantial concern in recent years. Pulmonary involvement has been frequently reported in cases with underlying disease, especially in cystic fibrosis (CF) (Dunne and Maisch, 1995; Ferroni et al., 2002). In CF patients, *A. xylosoxidans* isolated from sputum increased gradually (Zemel et al., 2000; Liu et al., 2002). However, CF is a rare disease in China. In non-CF patients, several cases of pneumonia have been reported in patients with underlying malignancy (Aisenberg et al., 2004), those with IgM deficiency (Dworzack et al., 1978) and those on mechanical ventilation (Chandrasekar et al., 1986). Additionally, approximately half of these non-CF patients originated from the community, which may be related to the soil and aquatic environments (Swenson and Sadikot, 2015). However, HAP due to *Achromobacter* spp. in the elderly is rare.

The features of this organism in the elderly need to be urgently studied. Therefore, this study aimed to understand the clinical characteristics, epidemiology, biofilm production and integrons of *Achromobacter* spp. in elderly patients with HAP.

## Materials and Methods

### Patients

Fifteen patients with HAP due to *Achromobacter* spp. treated in a geriatric ward in China from September 2008 to May 2012 were enrolled in this study. The diagnostic criteria of HAP were as described previously (Rotstein et al., 2007). Patient demographics and clinical characteristics (underlying disease, invasive manipulation, therapy, and mortality) were collected. The Acute Physiology and Chronic Health Evaluation II (APACHE II), Sequential Organ Failure Assessment (SOFA) score and Clinical Pulmonary Infection Score (CPIS) were evaluated within the first 24 h. Chronic infection was defined as a positive sputum culture on at least three occasions over a 6-month period, as previously suggested (Trancassini et al., 2014).

### Microbial Identification and *bla_OXA-*114*-like_* Analysis

*Achromobacter* spp. was cultured from sputum or aspirations more than twice and was the dominant species in the culture. All the strains were identified by the API 20 NE system and the Vitek II system as described previously (Otto-Karg et al., 2009; Tena et al., 2014). Species identification was confirmed by 16S ribosomal RNA gene sequencing (Mellmann et al., 2003). To further classify this organism, a specific PCR based on the detection of *bla_OXA-*114*-like_* was performed (Turton et al., 2011).

### Multilocus Sequence Typing

The primers and reaction conditions of seven housekeeping genes (*nusA*, *rpoB*, *eno*, *gltB*, *lepA*, *nuoL*, *nrdA*) were designed by searching Pubmlst^1^. Allelic profiling and ST determination were also performed through the above website. Additionally, phylogenetic analysis of housekeeping genes and eBURST analysis were performed. The concatenation of the seven housekeeping genes (total of 2249 bp) of *Achromobacter* spp. was performed. A dendrogram was constructed from the concatenated sequences by the neighbor-joining method (MEGA 6.05). The eBURST analysis was conducted using eBURST V3.

### Biofilm Production Assay

All isolates were individually cultured overnight in M-H medium (OXOID). A monoclonal sample from the M-H medium was mixed with LB medium to prepare a bacterial suspension of 0.5 units. Bacterial suspensions (200 μl) were inoculated in a 96-well plate followed by 48 h of incubation at 37°C in a bacterial incubator (German He Liman). Plankton bacteria were removed by washing three times with distilled water every 30 s. The wells were dyed at room temperature for 5 min and then incubated with 200 μl of 1% Crystal Violet dye for 20 min. They were then washed twice with distilled water and dried at 37°C for 30 min. Two hundred microliters of 95% ethanol was added into each well, and the optical density (OD) at 570 nm was measured in a microtiter auto reader (Thermo Fisher Scientific, US) (Trancassini et al., 2014; Zhang et al., 2016). ATCC *Escherichia coli* DH5a (Ac) was used as a negative control. The established criteria (four levels of biofilm-forming ability) were the following: (1) N: no biofilm producer (-), A ≤ Ac; (2) W: weakly positive (++), Ac < A ≤ 2Ac; (3) M: moderately positive (++), 2Ac < A ≤ 4Ac; and (4) S: strongly positive (+++), A > 4Ac (Stepanović et al., 2007).

### Susceptibility Testing

Antimicrobial susceptibility testing was conducted using the microbroth dilution method as described previously (Yamamoto et al., 2012). The etest was employed for polymyxin B in M-H medium (OXOID) (**Table 1**). The results were interpreted using the 2015 Clinical and Laboratory Standards Institute (CLSI) guidelines.

**Table 1:** The microbes feature of 15 cases.

NO.	AN	GM	TOB	ATM	CAZ	FEP	CTX	CRO	CPZ	LEV	CIP	IPM	MEM	TCY	PB	PIP	TZP	SXT	F	Int.	VR	*bla_OXA-*114*_*	ST	BF
1	>64	>16	>16	>64	4	>64	>64	>64	8	4	4	16	4	8	4	<16	4/2	<2/38	>512	I	*aadB + aadA2*	*bla_OXA-*114q*_*	307	W
2	>64	>16	>16	>64	>32	>64	>64	>64	32	2	2	2	1	2	1	<16	4/4	>4/76	256	I	*dfrA17 + aacA4*	*bla_OXA-*114q*_*	306	M
3	>64	>16	>16	>64	4	32	>64	>64	4	1	0.5	4	2	8	1	<16	4/2	<2/38	>512	I	*aadB + aadA2*	*bla_OXA-*114h*_*	5	W
4	>64	>16	>16	>64	16	>64	>64	>64	32	2	>4	8	0.5	>16	2	<16	4/4	<2/38	>512	I	*aadB + aadA2*	*bla_OXA-*114q*_*	306	W
5	>64	>16	>16	>64	>32	>64	>64	>64	>64	>8	>4	4	8	>16	4	<16	4/4	>4/76	256	I	*aadB*	*bla_OXA-*114h*_*	5	M
6	>64	>16	>16	>64	4	>64	>64	>64	4	4	>4	2	2	8	2	<16	4/2	<2/38	>512	I	*aadB*	*bla_OXA-*114r*_*	311	W
7	>64	>16	>16	>64	8	>64	>64	>64	8	4	4	16	4	8	1	<16	4/4	<2/38	>512	I	*aadB + aadA2*	*bla_OXA-*114q*_*	312	W
8	>64	>16	>16	>64	16	>64	>64	>64	32	4	>4	64	>128	>16	8	64	64/4	<2/38	256	I	*aadB + aadA2*	*bla_OXA-*114r*_*	313	S
9	>64	>16	>16	>64	8	>64	>64	>64	32	4	2	8	8	8	2	128	128/4	<2/38	>512	I	*aadB + aadA2*	*bla_OXA-*114h*_*	5	M
10	>64	>16	>16	>64	16	>64	>64	>64	32	4	>4	>128	>128	>16	16	32	32/4	<2/38	>512	I	*aadB + aadA2*	*bla_OXA-*114t*_*	314	S
11	>64	>16	>16	>64	>32	32	>64	>64	8	>8	>4	64	64	2	32	<16	4/2	>4/76	>512	I	*aadB + aadA2*	*bla_OXA-*114q*_*	306	S
12	>64	>16	>16	>64	8	>64	>64	>64	>64	2	2	16	4	8	2	<16	4/4	<2/38	256	I	*aadB + aadA2*	*bla_OXA-*114q*_*	306	M
13	>64	>16	>16	>64	8	>64	>64	>64	4	8	>4	4	4	>16	2	<16	4/4	<2/38	>512	I	*aadB + aadA2*	*bla_OXA-*114q*_*	5	S
14	>64	>16	>16	>64	8	>64	>64	>64	64	8	4	4	4	4	8	128	128/4	<2/38	>512	I	*blaPSE-1 + aac(6′)-II + aadB*	*bla_OXA-*114q*_*	306	M
15	>64	>16	>16	>64	>32	>64	>64	>64	>64	8	4	>128	>128	>16	2	<16	8/4	<2/38	>512	I	*aadB + aadA2*	*bla_OXA-*114r*_*	313	M

*AN, amikacin; GM, gentamicin; ATM, aztreonam; CAZ, ceftazidime; FEP, cefepime; CTX, cefotaxime; CRO, ceftriaxone; CPZ, cefoperazone; LEV, levofloxacin; CIP, ciprofloxacin; IPM, imipenem; MEM, meropenem; TCY, tetracycline; PB, polymyxin B; PIP, piperacillin; TZP, piperacillin/tazobactam; SXT, trimethoprim/sulfamethoxazole; F, nitrofurantoin; VR, variable region; ST, sequence typing; BF, bacterial biofilm.*

#### *Bla_OXA-*114*-like_* Analyses

The dendrogram analysis of *bla_OXA-*114*-like_* was performed using MEGA (version 6.05). The alignment used for the tree calculation and various amino acid sequences of OXA enzymes were performed with Clustal X (version 1.83). To determine whether different subtypes of *bla_OXA-*114*_* relate to different antibacterial spectra, antibacterial spectra were compared among these subtypes.

### PCR

Total DNA was prepared and used as a template for PCR reactions. PCR screening for integrase (*intI1*, *intI2*, and *intI3*) genes was performed as described previously (Yamamoto et al., 2012). The primers used in this study are shown in Supplementary Table S1 (Woodford et al., 2006; Poirel et al., 2011; El Salabi et al., 2012; Poirel et al., 2012).

The primers designed for various regions were Pf: GCATCCAAGCAGCAAG and Pr: AAGCAGACTTGACCTGA. PCR was performed using the Pyrobest DNA Polymerase according to the manufacturer’s instructions (Takara). PCR products were resolved on 1.0% agarose gels, stained with ethidium bromide, and photographed with UV illumination. The PCR products were then sequenced after purifying the DNA by using the Gel Extraction Kit according to the manufacturer’s directions (Axygen). The products were sequenced by Major Biological Medicine Technology Co Ltd in Shanghai.

### Nucleotide Sequence Accession Numbers

**FIGURE 1:**
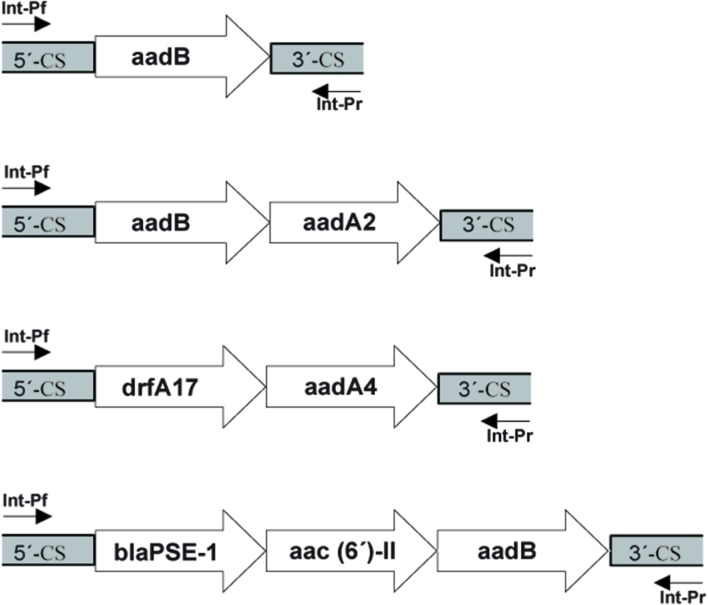
**Schematic representation of the class 1 integron gene cassettes in *Achromobacter* spp. isolates**.

The nucleotide sequences of the various regions in the integrons reported in this study were submitted to the NCBI^2^ and assigned the accession numbers KT454532, KT719385, KT719386, and KT719387 (**Figure 1**).

## Results

### Clinical Characteristics

These infections first occurred in 2008, and 2, 3, 5, 1, and 3 infections happened per year from 2008 to 2012, respectively. Patient characteristics are summarized in **Table 2** and Supplementary Table S2. In total, 14 males and 1 female were included in this study. The mean age was 88.8 ± 5.4 years. All the patients had more than 3 diseases, and 10 of them had 3 or more catheters. The primary underlying diseases were coronary heart disease (CHD) and chronic pulmonary disease. Eleven patients received proton pump inhibitors (PPI), three patients received corticosteroids and two received chemotherapy. Almost all these patients were treated with various antimicrobial therapies within the previous 14 days. Chest imaging of all patients showed patchy exudation. Pleural effusion was found in 33.3% (5/15) of cases, and one case showed consolidation. The mean APACHE II, SOFA and CPIS scores were 23.4 ± 7.7, 7.4 ± 2.7, and 7.6 ± 1.64, respectively. Twelve patients were given a different antibiotic regimen after the antimicrobial susceptibility test. Nine patients were treated by mechanical ventilation. Three patients had chronic infections lasting more than 1 year. Of those with infections, 10 were alive, and 5 (33.3%) died 30 days after infection. The fatal cases were more likely to have sepsis (*n* = 3).

**Table 2:** Detailed clinical features of HAP with *Achromobacter* spp. infection.

No.	Sex/Age	Comorbidities	Tube	Predisposing factor	Antibiotic use in 14 days	Clinical presentation	Empiric therapy	Switched therapy	Mechanical ventilation	APACHE II	CPIS	SOFA	Compli cations	Outcome at 30 days	Chest Imaging
1	M/88	CHD; Chronic bronchitis; Hypertension; Malignancy	CVC	PPI; Corticosteroids; Chemotherapy	TZP; Voriconazale; ONZ; IPM; Ceftriaxonze;	Fever(Tmax38.4°C); Cough; Purulent Sputum	LEV	MEM	Non	17	9	6	RF	Survived	Exudation(R); consolidation (L)
2	M/89	COPD;OPT; Hypertension; Arrhythmia;	CVC; Ureter; Stomach tube; Tracheostomy cannula	PPI	MIN;TZP;IPM	Fever(Tmax38.5°C) Purulent Sputum	SCF	MEM	Invasive	25	11	11	RF	Survived	Bilateral exudation;
3	M/97	Cerebrovascular disease; Hypertension; CHD;Chronic bronchitis; Post operation;	Stomach tube; CVC; Tracheostomy cannula	Non	Non	Purulent Sputum	TZP	TZP	Non	15	6	5	Non	Survived	Bilateral exudation; Pleural effusion(L)
4	M/75	ILD;CHD; Diabetes; CRF;Chronic bronchitis; Hypertension; Cerebrovascular disease; Arrhythmia;	CVC; Ureter; Stomach tube; Tracheostomy cannula	PPI	TZP, SXT, MXF, Linezolid; Voriconazole	Fever(Tmax38.0°C); Purulent Sputum	SCF	MEM	Non	28	8	9	RF	Survived	Exudation(R)
5	M/88	CHD; CRF; Diabetes Hypertension; Arrhythmia;	CVC; Ureter; Stomach tube; Nasotracheal intubation	PPI	MEM; CAZ; MNZ; Caspofungin	Fever(Tmax38.0°C); Chill; Cough; Purulent Sputum	LEV	TZP+MEM	Non	32	10	7	Sepsis	Died	Bilateral exudation;
6	M/85	CHD; Diabetes; Chronic bronchitis; Hypertension; Cerebrovascular disease;	Stomach tube	Non	Cefditoren pivoxil	Purulent Sputum	MXF	MEM	Non	13	5	4	Hyoxemia	Survived	Exudation(L)
7	M/95	ILD; CHD; Hypertension; Arrhythmia; Diabetes; Cerebrovascular disease;	CVC; Stomach tube	PPI	Non	Dyspnea; Cyanosis; Cough; Purulent Sputum	FMOX	MXF	Non	13	6	9	RF	Survived	Bilateral exudation
8	M/87	CHD; Hypertension; Arrhythmia	CVC; Ureter; Stomach tube; Tracheostomy cannula	PPI; Corticosteroids	MEM;MIN;SXT; FMOX;ONZ	Fever(Tmax38.0°C); Chill; Purulent Sputum	IPM	CAZ+TZP	Invasive	27	6	6	RF	Survived	Bilateral exudation; Pleural effusion(L)
9	M/93	Chronic bronchitis CHD; Diabetes Cerebrovascular disease	CVC; Ureter; Stomach tube; Tracheostomy cannula	Non	SCF;IPM; TZP; Linezolid; CIP; ONZ; Voriconazole; Caspofungin	Purulent Sputum	MXF	MEM+CAZ	Non-Invasive switch to Invasive	17	8	7	Sepsis shock	Died	Bilateral exudation; Bilateral effusion
10	M/93	COPD;CHD; CRF; Diabetes Hypertension; Cerebrovascular disease;	CVC; Ureter; Stomach tube; Tracheostomy cannula	PPI	MXF; Linezolid; Voriconazole	Fever(Tmax38.5°C); Chill; Purulent Sputum	IPM	CAZ+TZP	Invasive	35	8	12	Sepsis	Died	Bilateral exudation
11	F/82	Chronic bronchitis;CHD; Diabetes; Cerebrovascular disease	CVC; Ureter; Stomach tube; Tracheostomy cannula	Non	LEV;SCF;MEM; Caspofungin	Fever(Tmax38.2°C); Purulent Sputum	IPM	TZP+MIN	Invasive	18	7	7	ARDS	Died	Bilateral exudation; Bilateral effusion
12	M/89	CHD; ILD; Chronic bronchitis; Malignancy; Cerebrovascular disease	CVC; Stomach tube	PPI; Chemotherapy	Etimicin;	Purulent Sputum	MEM	MEM	Non-Invasive	36	8	10	MOF	Died	Exudation(R); Pleural effusion(R)
13	M/92	CHD;OPT; Chronic bronchitis; Hypertension; Cerebrovascular disease	Stomach tube	PPI	MEM;TZP;MIN; Caspofungin	Fever(Tmax37.8°C); Purulent Sputum	IPM	IPM	Invasive	21	6	4	Non	Survived	Exudation(R)
14	M/89	COPD;ILD; CHF Cerebrovascular disease	CVC; Stomach tube; Tracheostomy cannula	PPI	FMOX	Purulent Sputum	SCF	IPM+MIN	Non-Invasive switch to invasive	26	8	9	RF	Survived	Bilateral exudation
15	M/90	COPD;CHD; CRF; Diabetes Hypertension; Arrhythmia; Cerebrovascular disease;	CVC; Ureter; Stomach tube; Tracheostomy cannula	PPI; Corticosteroids	MXF; Linezolid, Voriconazole	Purulent Sputum	IPM	TZP	Non-Invasive	28	8	7	RF	Survived	Bilateral exudation

*CHD, coronary heart disease; COPD, chronic obstructive pulmonary disease; OPT, obsolete pulmonary tuberculosis; ILD, interstitial lung disease; CRF, chronic renal failure; CHF, congestive heart failure; RF, respiratory failure; ARDS, acute respiratory distress syndrome; MOF, multiple organ failure; CVC, central venous catheter; PPI, proton-pump inhibitor; TZP, piperacillin/tazobactam; ONZ, ornidazole; IPM, imipenem; MNZ, metronidazole; MEM, meropenem; SXT, trimethoprim/sulfamethoxazole; MXF, moxifloxacin; CAZ, ceftazidime; MNZ, metronidazole; MIN, minocycline; FMOX, flomoxef; SCF: cefoperazone/sulbactam; CIP, ciprofloxacin; LEV, levofloxacin; FEP, cefepime, R, right, L, left.*

### Diversity of *bla_OXA-*114*-like_*

**FIGURE 2:**
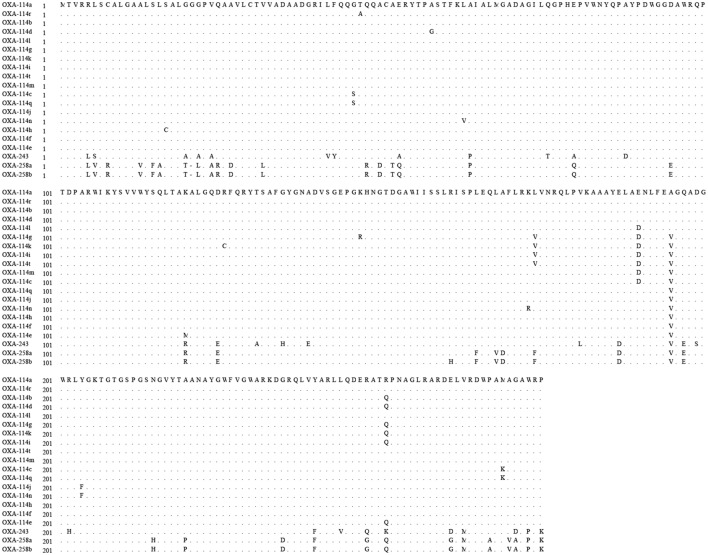
**Amino acid sequence differences in the OXA enzymes described in this study**.

**FIGURE 3:**
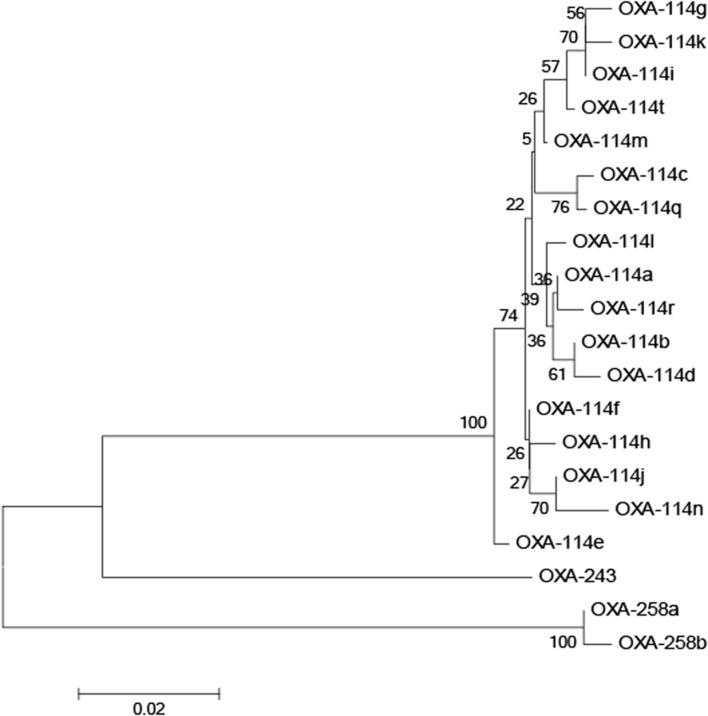
**Dendrogram analysis of OXA enzymes in *Achromobacter* spp. isolates**. The tree was inferred for various *bla_OXA-*114*_* types, *bla_OXA-*243*_*, and *bla_OXA-*253*_* using the neighbor-joining method.

All *Achromobacter* spp. strains had positive results for the amplification of *bla_OXA-*114*-like_* (**Figure 2**). To further study the sequence diversity of *bla_OXA-*114*-like_* in *Achromobacter* spp., amino acid sequence comparison of the *bla_OXA-*114*-like_* gene was conducted and an evolutionary tree were constructed (**Figure 3**). The most frequent type was *bla_OXA-*114*q_*, which accounted for 53.3% (8/15) of isolates. Other genes included *bla_OXA-*114*r_* (three cases), *bla_OXA-*114*h_* (three cases) and *bla_OXA-*114*t_* (one case). There were amino acid differences among all of the isolates.

### MLST and BF

**FIGURE 4:**
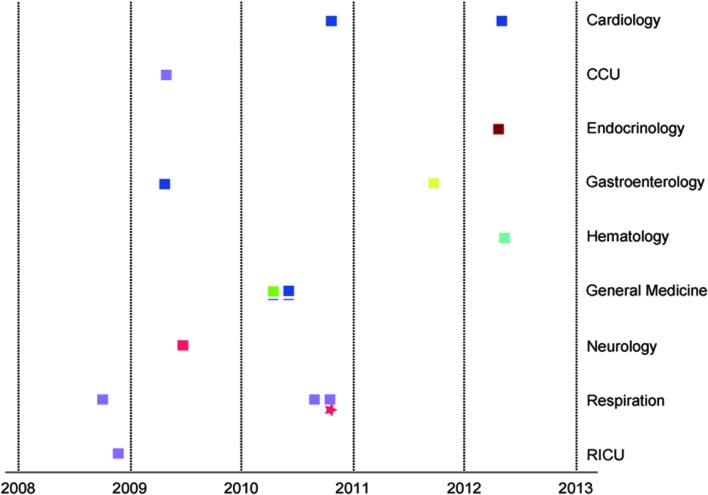
**The distribution of the 15 patients by admission department and year. 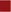: ST311 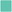:ST307 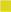:ST312 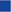:ST5 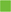:ST314 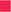:ST313 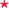:ST313 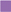:ST306.** CCU, cardiac intensive care unit; RICU, respiratory intensive care unit. General Medicine (the blue underline) is in another building. The same sequence type is shown in the same color square. The square represents male gender. The star represents female gender.

**FIGURE 5:**
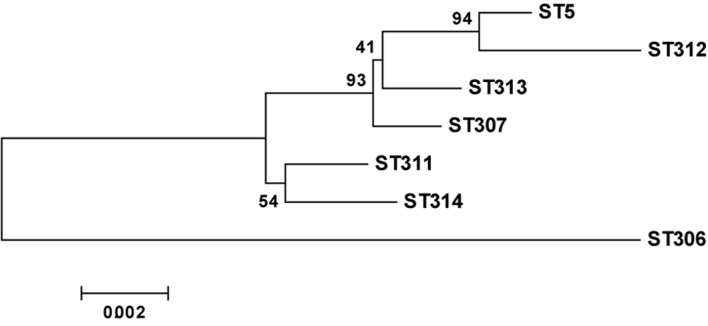
**Neighbor-joining dendrogram of concatenated sequences of seven housekeeping genes from the MLST database**.

**FIGURE 6:**
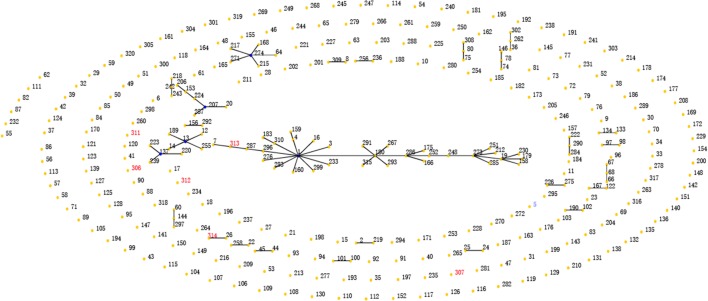
**eBRUST analysis of all STs of *Achromobacter***. Circles represent different STs; blue represents a primary founder, yellow represents a subgroup founder, red denotes STs found in the present study dataset only.

Based on MLST, four cases belonged to ST5, and these cases were distributed in two separate buildings of the hospital. Six new STs were detected (ST306, 307, 311, 312, 313, 314). Five cases belonged to ST306, and 2 belonged to ST313 (**Figure 4**). Phylogenetic analysis of housekeeping genes showed that there was a less obvious relationship between ST5 and ST314 (**Figure 5**), which were found in cases from the general medicine department in a different building from where the other STs were isolated. Additionally, all STs available in the database were analyzed via eBURST. The results show that ST313 belongs to the ST1 CC (clone complex), and HAP caused by this organism may occur sporadically (**Figure 6**).

All strains were able to produce a biofilm on 96-well plates (**Table 1**). Nearly half of the strains were moderate biofilm producers, four strains were strong producers, and five strains were weak producers.

### Antibiotic Resistance and Integron Variable Region

All strains were resistant to aminoglycosides, aztreonam, nitrofurantoin, and third- and fourth-generation cephalosporins (except ceftazidime and cefoperazone). Approximately half of these strains were resistant to ciprofloxacin, tetracycline and imipenem, but the sensitivity to piperacillin/tazobactam and meropenem was close to 70%.

**FIGURE 7:**
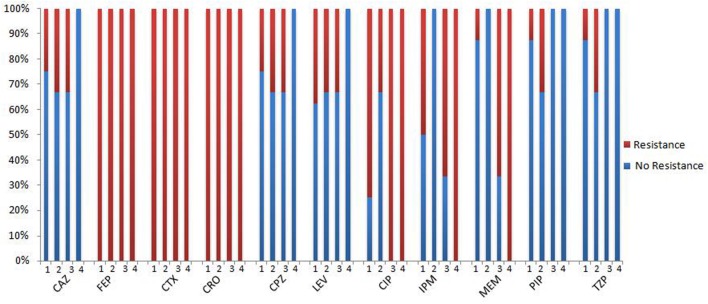
**The antibacterial spectrum of *bla_OXA-*114*-like_* -containing strains.** 1: *bla_OXA-*114q*_*, 2: *bla_OXA-*114q*_*, 3: *bla_OXA-*114q*,_* 4: *bla_OXA-*114q*_;* CAZ, ceftazidime; FEP, cefepime; CTX, cefotaxime; CRO, ceftriaxone; CPZ, cefoperazone; LEV, levofloxacin; CIP, ciprofloxacin; IPM, imipenem; MEM, meropenem; PIP, piperacillin; TZP, piperacillin/tazobactam.

All the *bla_OXA-*114*-like_* type strains showed high resistance to cefepime, cefotaxime and ceftriaxone and high sensitivity to piperacillin and piperacillin/tazobactam. It is interesting that both *bla_OXA-*114*t_* and *bla_OXA-*114*r_* strains are resistant to ciprofloxacin and show higher resistance to carbapenems than other subtypes (**Table 3**, **Figure 7**). However, due to the small sample, a large sample analysis should be conducted to further examine this finding.

**Table 3:** The antibacterial spectrum of *bla_OXA-*114*-like_* strain.

	Total	CAZ		FEP		CTX		CRO		CPZ		LEV		CIP		IPM		MEM		PIP		TZP	
	Number	NR	R	NR	R	NR	R	NR	R	NR	R	NR	R	NR	R	NR	R	NR	R	NR	R	NR	R
*bla_OXA-*114q*_*	8	6	2	0	8	0	8	0	8	6	2	5	3	2	6	4	4	7	1	7	1	7	1
*bla_OXA-*114h*_*	3	2	1	0	3	0	3	0	3	2	1	2	1	2	1	3	0	3	0	2	1	2	1
*bla_OXA-*114r*_*	3	2	1	0	3	0	3	0	3	2	1	2	1	0	3	1	2	1	2	3	0	3	0
*bla_OXA-*114t*_*	1	1	0	0	1	0	1	0	1	1	0	1	0	0	1	0	1	0	1	1	0	1	0

We examined three types of integrons *(intI*, *intII*, *intIII)*, and all the strains had *intI*, but neither *intII* nor *intIII* were found. In the variable region in *intI*, *aadB* + *aadA2* were detected in 10 strains, *aadB* was inserted in two strains, *dfrA17*+ *aacA4* in one isolate, and *bla_PSE-1_*+*aac(6′)-II* + *aadB* in one isolate (Supplementary Figure S1).

## Discussion

*Achromobacter* spp. have been frequently detected by the cystic pulmonary fibrosis (CF) center in recent years (Lambiase et al., 2011; Pereira et al., 2011; Amoureux et al., 2013b; Llorca Otero et al., 2016). A study showed that colonization mostly occurred in elderly patients who had more pronounced lung damage and poorer lung function (De Baets et al., 2007). Chronic pulmonary disease and respiratory tract infections often occur in elderly patients. In our study, elderly patients had more than three diseases, particularly CHD, chronic pulmonary disease and cerebrovascular disease. Additionally, 10 had 3 or more indwelling catheters. These catheters may contribute to the higher APACHE II and SOFA scores. The mortality rate was 33.3% (5/15), which is higher than that in other studies (Gómez-Cerezo et al., 2003; Aisenberg et al., 2004). Piperacillin/tazobactam and meropenem showed a strong ability to treat the infection, consistent with the *in vitro* susceptibility tests. In our study, nine patients (60%) received mechanical ventilation. This organism can colonize humidifiers, which is an issue that needs to be addressed when a patient receives mechanical ventilation (Tena et al., 2005).

More than 300 types of *Achromobacter* spp. have been recorded in the MLST database. ST5 was first cultured from a CF patient in USA. In our hospital, four cases were infected by this type from 2009 to 2012 in two buildings. These patients did not appear to be clustered in space or time. Moreover, the MIC of carbapenems for ST5 was lower. From 2008 to 2010, ST306 was detected in the Respiration and CCU. Interestingly, the same ST showed various antibiotic-resistance spectra, indicating different resistance mechanisms. The other STs (ST307, ST311, ST312, ST313, and ST314) did not appear to be clustered in space or time. Additionally, the location and timing were not consistent with the six non-clustered cases. However, no strain was detected from doctors’ hands or from medical equipment. The source of their infection could not be determined. Two strains (ST313) showed higher MIC for cephalosporins, quinolones and carbapenems, which should be considered in clinical practice.

It has been proposed that *bla_OXA-*114*-like_* is an inherent gene for *Achromobacter xylosoxidans* (Turton et al., 2011). Additionally, the diversity of *bla_OXA-*114*-like_* has been reported (Amoureux et al., 2013a). *Bla_OXA-*114*q_*, *bla_OXA-*114*r_*, *bla_OXA-*114*h_* and *bla_OXA-*114*t_* were also detected in our study, which confirmed the diversity of this gene. We have listed the similarities and differences in amino acids among these genes in our isolates. Further studies are needed to understand the function of these differences.

In our study, the strains isolated from patients who were supported by mechanical ventilation had stronger biofilm production. Additionally, the strong biofilm producers (No. 8, 10, 11) showed higher MIC of carbapenems. These characteristics may be shared by other Gram-negative biofilm producers.

Various regions of integrons can carry various resistance genes. In Argentina, class 1 integrons and class 2 integrons were detected in an *Achromobacter* spp., including various antibiotic-resistant genes (Traglia et al., 2012). In the present study, only class 1 integrons were detected, and all strains were resistant to aminoglycosides, consistent with the three types of genes (*aadB*, *aadA2* and *aacA4*) detected in the integron.

The overuse of antibiotics may be a reason for the increasing opportunistic infections with resistant microorganisms (Tan et al., 2002). *In vitro*, these isolates showed high sensitivity to trimethoprim/sulfamethoxazole, polymyxin B, meropenem and piperacillin/tazobactam. In clinical practice, due to the hepatic and renal toxicity of trimethoprim/sulfamethoxazole and polymyxin B, meropenem and piperacillin/tazobactam are frequently used in therapy. Additionally, in our study, nine patients survived after meropenem and piperacillin/tazobactam therapy. However, four isolates showed high resistance to imipenem *in vitro*. In a previous study, the *bla_VIM_* located in integron I was reported in both Asia and Europe (Riccio et al., 2001; Shin et al., 2005; Sofianou et al., 2005). Additionally, a new type of *bla_IMP_* inserted in plasmids was detected in *A. xylosoxidans*, which could easily trigger a pandemic of carbapenem-resistant isolates (Yamamoto et al., 2012). Unfortunately, metallo-b-lactamase (MBL) gene cassettes involved in carbapenem resistance were not detected in the integrons. Other mechanisms may account for the carbapenem resistance observed in this study. Thus, whole genome sequencing may be needed for further study.

Many studies showed that *dfrA* was frequently detected in integrons (Yu et al., 2003, 2004). In our study, *dfrA17* was found in integron I, which was once detected in patients with urinary tract infection from another Gram-negative organism in Korea. However, the clinical specimens of our patients were sputum samples. The spread of *dfrA17* may be related to the plasmid (Yu et al., 2003). The *bla_PSE-*1*_* gene was first reported in *Achromobacter* spp. This gene can induce carbenicillin-hydrolyzing beta-lactamases aimed at carboxypenicillins, ureidopenicillins and cefsulodin. These enzymes belong to molecular class A and functional group 2c (Bush et al., 1995), which can be frequently detected in Gram-negative organisms. These results indicate that this organism may be a reservoir of horizontal genetic transfer elements for spreading antibiotic resistance (Collis and Hall, 1995).

This study has several limitations. First, it is a small retrospective study conducted over 5 years. Therefore, the source of infection could not be determined, and our results cannot be generalized to other populations. Second, these cases of *Achromobacter* spp. in China are not routinely reported. Third, the mechanism of resistance to carbapenems was unclear. Whole genome sequencing may be needed in further studies.

## Author Contributions

XF: study design, data collection, data analysis, writing of the manuscript; CL: study design, data collection and analysis, writing of the manuscript. Chao L: study design, data collection and analysis, writing of the manuscript; FP: data collection and analysis, writing of the manuscript; JG: data analysis, writing of the manuscript; YJ: data analysis, writing of the manuscript; LQ, YJ, and WY: data collection; All authors have read and approved the final version of the manuscript.

## Conflict of Interest Statement

The authors declare that the research was conducted in the absence of any commercial or financial relationships that could be construed as a potential conflict of interest.

## Footnotes

1 http://pubmlst.org/achromobacter/

2 http://www.ncbi.nlm.nih.gov/
