# Supplementary material for: Hospital Acquired Pneumonia Due to Achromobacter spp. in a Geriatric Ward in China: Clinical Characteristic, Genome Variability, Biofilm Production, Antibiotic Resistance and Integron in Isolated Strains
Source: Front Microbiol. 2016 May 9;7:621. doi: 10.3389/fmicb.2016.00621 (PMC4860489; doi:10.3389/fmicb.2016.00621)
Supplement: Supplementary file 1 [file Table_1.DOCX]

**Supplementary data**

**Table S1. Primers used in this study.**

| Gene Target | Primer name | Primer sequence | References |
| --- | --- | --- | --- |
| int I | int I-F  int I-R | GTTCGGTCAAGGTTCTGG CGTAGAGACGTCGGAATG | This study |
| int II | int II-F  int II-R | CAAGCATCTCTAGGCGTA  AGAAGCATCAGTCCATCC | This study |
| int III | int III -F  int III -R | CATCAAGCTGCTCGATCA  ACAACTCTTGCACCGTTC | This study |
| *bla_IMP_* | IMP-F  IMP-R | GGAATAGAGTGGCTTAAYTCTC  GGTTTAAYAAAACAACCACC | [19] |
| *bla_VIM_* | VIM-F  VIM-R | GATGGTGTTTGGTCGCATA  CGAATGCGCAGCACCAG | [19] |
| *bla_NDM_* | NDM-F  NDM-R | GGTTTGGCGATCTGGTTTTC  CGGAATGGCTCATCACGATC | [[19](#_ENREF_2)] |
| *bla_SPM_* | SPM-F  SPM-R | AAAATCTGGGTACGCAAACG  ACATTATCCGCTGGAACAGG | [19] |
| *bla_GIM_* | GIM-F  GIM-R | TCGACACACCTTGGTCTGAA  AACTTCCAACTTTGCCATGC | [19] |
| *bla_SIM_* | SIM-F  SIM-R | TACAAGGGATTCGGCATCG  TAATGGCCTGTTCCCATGTG | [19] |
| *bla_TMB_* | TMB-F  TMB-R | CAAGGAGCTCATTCAAAGG  TTCTAGCGGATTGTGGCCAC | [[20](#_ENREF_3)] |
| OXA-23-like | OXA-23-F  OXA-23-R | GATCGGATTGGAGAACCAGA  ATTTCTGACCGCATTTCCAT | [[21](#_ENREF_6)] |
| OXA-24-like | OXA-24-F  OXA-24-R | GGTTAGTTGGCCCCCTTAAA  AGTTGAGCGAAAAGGGGATT | [[21](#_ENREF_6)] |
| OXA-48-like | OXA-48-F  OXA-48-R | TTGGTGGCATCGATTATCGG  GAGCACTTCTTTTGTGATGGC | [[22](#_ENREF_7)] |
| OXA-58-like | OXA-58-F  OXA-58-R | AAGTATTGGGGCTTGTGCTG  CCCCTCTGCGCTCTACATAC | [[21](#_ENREF_6)] |
| OXA-114 | OXA114-Pf | CGCATCCTGTTCCAGCA | This study |
|  | OXA114-Pr | GTGCCGGTCTTGCCATAC |  |
| VR | VR-F  VR-R | GGCATCCAAGCAGCAAG  AAGCAGACTTGACCTGA | This study |
