# Supplementary material for: Hospital Acquired Pneumonia Due to Achromobacter spp. in a Geriatric Ward in China: Clinical Characteristic, Genome Variability, Biofilm Production, Antibiotic Resistance and Integron in Isolated Strains
Source: Front Microbiol. 2016 May 9;7:621. doi: 10.3389/fmicb.2016.00621 (PMC4860489; doi:10.3389/fmicb.2016.00621)
Supplement: Supplementary file 2 [file Table_2.DOCX]

**Supplementary data**

Table S2. Clinical features of HAP with *Achromobacter.spp* infection.

| Item | Total number | Number of positive cases | Positive rate (% ) |
| --- | --- | --- | --- |
| **Gender, male** | 15 | 14 | 93.3 |
| **Age, years, average±SD(range)** | | 88.8±5.4 |  |
| **Comorbidities** |  |  |  |
| Coronary Heart Disease | 15 | 13 | 86.7 |
| Cerebrovascular disease | 15 | 11 | 73.3 |
| Hypertension | 15 | 11 | 73.3 |
| Chronic bronchitis | 15 | 8 | 53.3 |
| Diabetes | 15 | 8 | 53.3 |
| Arrhythmia | 15 | 6 | 40.0 |
| Chronic Obstructive Pulmonary Disease | 15 | 4 | 26.7 |
| Chronic Renal Failure | 15 | 4 | 26.7 |
| Interstitial Lung Disease | 15 | 4 | 26.7 |
| Malignancy | 15 | 2 | 13.3 |
| Obsolete Pulmonary Tuberculosis | 15 | 2 | 13.3 |
| Post operation | 15 | 1 | 6.7 |
| Congestive Heart Failure | 15 | 1 | 6.7 |
| **Tube** |  |  |  |
| Stomach tube | 15 | 14 | 93.3 |
| Central vena catheterization | 15 | 13 | 86.7 |
| Tracheostomy cannula | 15 | 9 | 60.0 |
| Ureter | 15 | 8 | 53.3 |
| Nasotracheal intubation | 15 | 1 | 6.7 |
| **Predisposing Factor** |  |  |  |
| Proton-pump inhibitor | 15 | 11 | 73.3 |
| Corticosteroids | 15 | 3 | 20.0 |
| Chemotherapy | 15 | 2 | 13.3 |
| **Antibiotic use in 14 days** |  |  |  |
| Aminoglycosides | 15 | 1 | 6.7 |
| Etimicin | 15 | 1 | 6.7 |
| Cephalosporins | 15 | 5 | 33.3 |
| Ceftazidime | 15 | 1 | 6.7 |
| Cefditoren pivoxil | 15 | 1 | 6.7 |
| Ceftriaxonze | 15 | 1 | 6.7 |
| Flomoxef | 15 | 2 | 13.3 |
| Quinolones | 15 | 5 | 33.3 |
| Ciprofloxacin | 15 | 1 | 6.7 |
| Levofloxacin | 15 | 1 | 6.7 |
| Moxifloxacin | 15 | 3 | 20.0 |
| Carbapenems | 15 | 7 | 46.7 |
| Imipenem | 15 | 3 | 20.0 |
| Meropenem | 15 | 4 | 26.7 |
| Tetracyclines | 15 | 3 | 20.0 |
| Minocycline | 15 | 3 | 20.0 |
| β-Lactamaseinhibitors | 15 | 7 | 46.7 |
| Cefoperazone/Sulbactam | 15 | 2 | 13.3 |
| Piperacillin/Tazobactam | 15 | 5 | 33.3 |
| Sulfonamide | 15 | 2 | 13.3 |
| Trimethoprim/sulfamethoxazole | 15 | 2 | 13.3 |
| Oxazolidinone antibiotic | 15 | 4 | 26.7 |
| Linezolid | 15 | 4 | 26.7 |
| Antifungal drug | 15 | 9 | 60.0 |
| Caspofungin | 15 | 4 | 26.7 |
| Voriconazole | 15 | 5 | 33.3 |
| Nitroimidazoles | 15 | 4 | 26.7 |
| Metronidazole | 15 | 1 | 6.7 |
| Ornidazole | 15 | 3 | 20.0 |
| Non | 15 | 2 | 13.3 |
| **Clinical Presentation** |  |  |  |
| Purulent Sputum | 15 | 15 | 100.0 |
| Fever | 15 | 8 | 53.3 |
| Chill | 15 | 3 | 20.0 |
| Cough | 15 | 3 | 20.0 |
| Cyanosis | 15 | 1 | 6.7 |
| Dyspnea | 15 | 1 | 6.7 |
|  |  |  |  |
| **Empiric Therapy** |  |  |  |
| Cephalosporins | 15 | 1 | 6.7 |
| Flomoxef | 15 | 1 | 6.7 |
| Carbapenems | 15 | 6 | 40.0 |
| Meropenem | 15 | 1 | 6.7 |
| Imipenem | 15 | 5 | 33.3 |
| Quinolones | 15 | 4 | 26.7 |
| Levofloxacin | 15 | 2 | 13.3 |
| Moxifloxacin | 15 | 2 | 6.7 |
| β-Lactamaseinhibitors | 15 | 4 | 26.7 |
| Cefoperazone/sulbactam | 15 | 3 | 20.0 |
| Piperacillin/Tazobactam | 15 | 1 | 6.7 |
| **Switched Therapy** |  |  |  |
| Moxifloxacin | 15 | 1 | 6.7 |
| Meropenem | 15 | 5 | 33.3 |
| Meropenem+ Ceftazidime | 15 | 1 | 6.7 |
| Imipenem | 15 | 1 | 6.7 |
| Imipenem+ Minocycline | 15 | 1 | 6.7 |
| Piperacillin/Tazobactam | 15 | 2 | 13.3 |
| Piperacillin/Tazobactam+ Ceftazidime | 15 | 2 | 13.3 |
| Piperacillin/Tazobactam+ Meropenem | 15 | 1 | 6.7 |
| Piperacillin/Tazobactam+ Minocycline | 15 | 1 | 6.7 |
| **Mechanical Ventilation** |  |  |  |
| Non | 15 | 6 | 40.0 |
| Invasive | 15 | 5 | 33.3 |
| Non-Invasive | 15 | 2 | 13.3 |
| Non-Invasive switch to Invasive | 15 | 2 | 13.3 |
| **Complications** |  |  |  |
| Respiratory Failure | 15 | 7 | 46.7 |
| Non | 15 | 2 | 13.3 |
| Sepsis | 15 | 2 | 13.3 |
| Acute Respiratory Distress Syndrome | 15 | 1 | 6.7 |
| [Hyoxemia](javascript:void(0);) | 15 | 1 | 6.7 |
| Multiple Organ Failure | 15 | 1 | 6.7 |
| Sepsis shock | 15 | 1 | 6.7 |
| **Chest imaging** |  |  |  |
| Bilateral exudation | 15 | 10 | 66.7 |
| Unilateral exudation | 15 | 5 | 33.3 |
| Unilateral pleural effusion | 15 | 3 | 20.0 |
| Bilateral pleural effusion | 15 | 2 | 13.3 |
| Consolidation (L) | 15 | 1 | 6.7 |
| **APACHE II (mean± SD)** |  | 23.4±7.7 |  |
| **CPIS (mean± SD)** |  | 7.6±1.6 |  |
| **SOFA(mean± SD)** |  | 7.5±2.4 |  |
| **Mortality** | 15 | 5 | 33.3 |
