# Supplementary figures and images for: Hospital Acquired Pneumonia Due to Achromobacter spp. in a Geriatric Ward in China: Clinical Characteristic, Genome Variability, Biofilm Production, Antibiotic Resistance and Integron in Isolated Strains
Source: Front Microbiol. 2016 May 9;7:621. doi: 10.3389/fmicb.2016.00621 (PMC4860489; doi:10.3389/fmicb.2016.00621)

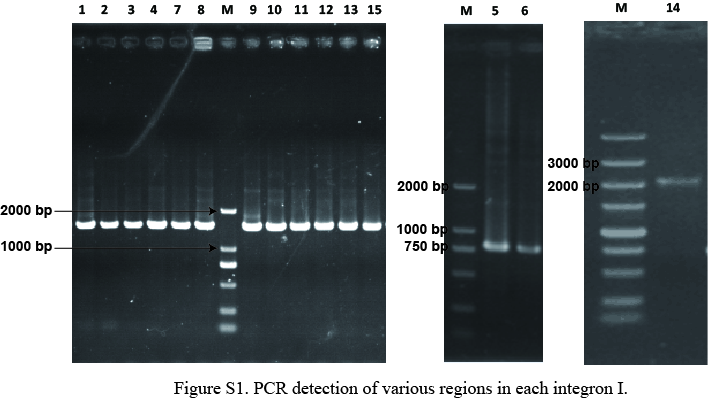

Supplement: Supplementary file 3 [file Image_1.TIF]
